# Multicentric Castleman's disease: a case report

**DOI:** 10.1186/1752-1947-1-78

**Published:** 2007-09-05

**Authors:** Brian F Menezes, Rosemary Morgan, Mohammed Azad

**Affiliations:** 1 Department of Medicine, University Hospital Aintree, Liverpool L9 7AL, UK; 2 Directorate of Medicine for the Elderly, Arrowe Park Hospital, Wirral CH49 5PE, UK

## Abstract

Castleman's disease is a clinicopathological entity associated with lymphoproliferation. We report a case of a 71 year old gentleman who was initially clinically suspected to have lymphoma (owing to clinical features at presentation), but was later histologically confirmed to have Castleman's disease. This case report underlines the importance of definitive histological diagnosis in patients with lympadenopathic presentation associated with systemic symptoms and the distinctiveness of multicentric Castleman's disease from malignant lymphoma. In this report we also attempt to provide new insight (through the review of medical literature) into the clinical features, pathogenesis, diagnosis and treatment of this rare and relatively benign disorder.

## Background

Castleman's disease(CD) is a heterogeneous group of lymphoproliferative disorders of uncertain cause [1] presenting with lymphadenopathy. It is histologically and prognostically distinct from malignant lymph-node hyperplasia. It was first described in a group of patients with benign localised hyperplastic lymph-nodes in 1956 by Castleman et al [2].

### Synonyms of Castleman's Disease

Angiofollicular Lymph-Node Hyperplasia, Giant Benign Lymphoma, Giant Lymph-Node Hyperplasia, Lymphoid Hamartoma

## Case presentation

A 71 year old gentleman was referred to the geriatric clinic of a district general hospital with a 2 month history of lethargy, decreased appetite and marked weight loss. He had no past medical history of note but had a brother who had died of lymphoma. Examination revealed mild bilateral cervical and axillary lymphadenopathy with no palpable organomegaly. Routine investigations such as full blood count and biochemical profile (including hepatic function tests) were found to be within normal range except for raised globulins (55 g/L) with a polyclonal increase in gamma-globulins. However, myeloma was excluded when serum protein electrophoresis detected no monoclonal band.

Computerised tomography revealed widespread lymphadenopathy involving the neck, axillae, chest/mediastinum, abdomen and pelvis with mild to moderate splenomegaly. Bone marrow aspiration and trephine biopsy showed small lymphoid follicular aggregates. Excision biopsy of an axillary node was performed and this was reported as a lymphoproliferative picture (increased number of follicles containing amorphous hyaline material and some small blood vessels) between simple reactive changes and frank lymphoma, suggesting Castleman's disease. He was referred to a haematologist and was commenced on steroid therapy which successfully induced disease remission and symptomatic relief.

## Discussion

Castleman's disease(CD) is lymphoproliferative disorder which is histologically characterised by angiofollicular lymph-node hypertrophy [3]. It may be borne in mind in the differential diagnoses of localised/diffuse lymphadenopathy with or without systemic manifestations. This case report, together with a review of medical literature in existence, attempts to provide new insight into this rather rare and relatively benign disorder which, though mimicking lymphoma clinically, varies from the latter histologically, prognostically and in its treatment options.

Localised CD is, by definition, localised to one site. It features lymphoid hyperplasia associated with excessive angiogenesis [1]. It is asymptomatic in over 50% of patients [4] and is often discovered incidentally. Histological diagnosis requires lymph-node biopsy.

Multicentric CD is characterized by a predominantly lymphadenopathic presentation consistently involving peripheral lymph-nodes and manifestations of multisystem involvement. It is considered as a systemic B cell lymphoproliferation, probably arising in immunoregulatory deficit, and resulting in the outgrowth of clonal B-cell populations [1]. It is always symptomatic. Symptoms, primarily a consequence of elevated Interleukin-6(IL-6) production, are asthenia(65%), weight loss(67%) and fever(69%) [3]. Polyadenopathy is common(84%) with a mean of four sites involved and is often associated with hepatosplenomegaly [3]. Histological diagnosis is made upon biopsy of an excised peripheral lymph-node.

A POEMS (**P**eripheral polyneuropathy, **O**rganomegaly, **E**ndocrinopathy, **M**onoclonal gammopathy(M-Protein) and **S**kin signs) syndrome [5] is observed in 24% of patients [3]. Some MCD forms are associated with Kaposi's sarcoma displaying prominent vascular proliferation and characteristic lesions. MCD associated with human immunodeficiency virus(HIV) infection is very similar to MCD observed in non-HIV-infected patients, except for the high prevalence of pulmonary symptoms and for the stronger association with Kaposi's sarcoma [6]. Progression to malignant lymphoma in MCD associated with HIV is frequent, and within a prospective cohort study [7] of 60 HIV-infected patients with MCD, and a follow-up period of 20 months, 14 patients(23%) developed HHV8-associated non-Hodgkin lymphoma.

The aetiology of Castleman's disease is poorly understood and no genetic or toxic factor has so far been identified. The hypothesis of a viral infection has been raised and several studies have suggested the role of human herpesvirus 8 (HHV-8), already implicated in Kaposi's sarcoma. In MCD, HHV-8 sequences were identified in 60–100% of patients infected with HIV and in 20–41% in those who were not [8,9]. These findings suggest two possibilities concerning the genesis of CD: (i) the opportunistic presence of HHV-8, favoured by immune pertubations; and (ii) the direct pathogenic role of HHV-8, in association with dysregulation of cytokines. Recent studies support the latter hypothesis by demonstrating that HHV-8 is able to produce an IL-6 homologue, the interleukin reponsible for the plasmacytosis and hypergammaglobulinaemia seen in MCD.

Localised CD is treated by surgical excision which allows full recovery without relapse in almost all cases. However, no therapeutic consensus exists for MCD and diverse treatments (surgery/corticotherapy/chemotherapy) are used, often in combination [3]. Anti-interleukin-6 antibody has also been successfully tried in the alleviation of systemic manifestations [10]. The five-year survival rate in MCD is 82% [3] and this prognosis appears to be far better than that encountered with malignant lymphomas.

## Conclusion

This case report brings to light the importance of obtaining definitive histological diagnosis in patients presenting with lymphadenopathy and systemic symptoms. Multicentric Castleman's disease is a relatively uncommon cause for such a presentation. Though clinically synonymous with lymphoma, it is an entity that is distinct from malignant lymphoproliferative disorders histologically and prognostically. It may be borne in mind as a differential diagnosis in lymphadenopathic presentations with symptoms of systemic involvement.

## Abbreviations

CD, Castleman's disease; MCD, multicentric Castleman's disease; HHV, human herpes virus; HIV, human immunodeficiency virus

## Consent

Written informed consent was obtained from the patient for the publication of this case report. A copy of the written consent is available for review by the Editor-in-Chief of this journal.

## Competing interests

The author(s) declare that they have no competing interests.

## Authors' contributions

Dr. Menezes made substantial contributions to the design, acquisition of data, literature review and drafting of the manuscript. Dr. Morgan and Dr. Azad were responsible for the conception, drafting and general supervision of this work. All authors have given final approval of the version to be published.
